# Concerning Dr. Howell’s Case

**Published:** 1891-02

**Authors:** E. H. Martin

**Affiliations:** Hillhouse, Miss.


### CONCERNING DR. HOWELL'S CASE.*

BY E. H. MARTIN, M. D., HILLHOUSE, MISS.

In the December number of the JOURNAL, I notice a report of a case of Hemorrhagic Malarial Fever (?), by Dr. J. R. G. Howell, of Dothan, Ala., in which he called particular attention to the presence of hemaglobin in the effusion of a blister. He draws deductions from this which are undoubtedly true—that, in cases of long continued malarial poisoning, the capillaries throughout the system, in the kidneys as well as in the integument, are so weakened as to allow the escape of the blood coloring matter upon the least increase of local blood pressure.

This is well shown by sucking the skin on the arm of such a subject—a bruise soon appears. But the capillaries are not alone to blame for this; the blood of such a patient also is very favorable to its dissolution.

But by far the most important lesson taught by his case, a lesson so plain that he who runs may read, and one which Dr. H., probably through a laudable orthodoxy, seems not to have fully learned, is the action of quinia in this disease. An uncertain dose of that drug produces so marked an increase in all of the bad symptoms that Dr. H. suspends the treatment *for a day*. Why not for a week?

Dr. H. will find this effect of quinia in every case of hemaglobinuria. The reason is plain to one who will think of the action of the drug. Given a patient whose constitution is run down below par by chronic malarial poisoning, whose capillaries are weakened and whose blood is in a state favorable to ready dissolution, administer a dose of quinia to such a patient. What is its effect—first on the blood? Bonoru and Arvedi, Magendie, Mouneret, Melier and Baldwin all found that the blood of animals killed with quinia was "dark, defibrinated, fluid and incapable of

*Published December, 1890, JOURNAL.

forming a clot." So, even a small dose given to such a patient must certainly add much to the already bad condition, and help prepare for what is to follow.

We have now but to direct the blood to a certain part, to produce a local increased blood pressure, and we will have an outpouring of blood coloring matter, as was seen in the blister and sucked spot on arm.

At this critical moment the quinia begins to be eliminated by the kidneys. That quinia influences these organs when in a healthy state is well known. Ranke first, then Kerner, and also Rabuteau, found that quinia decreased the elimination of urea uric acid, kreatinine and the collective nitrogeneous material. The decrease in urea was as great as forty per cent. Is it surprising, then, when the kidneys of such a patient are called on to bear this additional burden that the irritation, however slight, is the last straw ? The result is, malarial hematuria, hemorrhagic malarial fever, malarial hemaglobinuria, quinine hemaglobinuria, icterus, hematuric fever—call it what you may.

The truth of this is abundantly proven by practical experience —facts worth a thousand theories.

And that this condition should often last for several days after the ingestion of the last dose of quinia is not at all surprising when we consider that De Renzi found quinia in the urine of a *healthy* subject seven days after the last dose of the drug. Is it not extremely probable, that in this disease the alkaloid may remain even longer without elimination ?

Turpentine seems to hasten this elimination, and also appears to have some restorative or tonic influence on the kidneys which cannot be ascribed to its diuretic action alone. Be this action what it may, that it has such an influence is proven by many cases successfully treated all over the South.

That Dr. H.'s case did not terminate fatally was due to the amount of turpentine given and the decrease in the daily amount of quinia. We have here a singular condition—a patient alternately poisoned, with the best of motives and quinia, and in turn given an antidote ! Fortunately the relative amount of the antidote was greater than that of the poison ; and so the patient gradually improved and passed clear urine after seven days. Had the proportion been reversed the patient would have died—died from genuine poisoning. I know whereof I speak, for I successfully laid away a series of these patients in this way when I first began practice. I was ultra-orthodox in those days, followed the text-books implicitly, pushed quinia to its utmost and never let a case get well.

Had Dr. H. abandoned quinia, given the turpentine and iron as he did, also a mild cathartic or saline purgative and used arsenic as his antiperiodic, his patient's urine would have cleared up in from twelve to forty-eight hours, and the convalescence have been so much shorter. This statement also has been verified by myself and others by practical experience and this much dreaded disease been robbed of its horrors.

I will not claim that quinia is always the exciting cause of the disease, though I have never seen a case where quinia had not been taken previous to the attack. But I do believe that it always does harm in such cases, and for the above mentioned reasons. Now, I would not lead any one to be careful in the use of quinia ; one is too apt to be too careful and let cases suffer for want of it. We can never tell when or in which case the conditions are such as to forbid it until the attack gives warning. I use the drug constantly wherever indicated, but abandon it at once when the symptoms of hemaglobinuria appear.

If needed later on, quinia may be returned to ; the time when this can be done varies in different cases. It is as soon as patient's system—blood and capillaries especially—has been restored sufficiently to stand it.

I have in some cases given quinia within a week from clearing of urine, and have caused a return of the hemaglobinuria in only one case, in which the symptoms promptly disappeared on return to former treatment.

